# Glycosylation of Extracellular Vesicles: Analytical and Translational Insights into Biomarker Discovery and Regenerative Medicine

**DOI:** 10.3390/ijms27104298

**Published:** 2026-05-12

**Authors:** Muhammad Umair Khan, Ľuboš Danišovič, Jaroslav Katrlík

**Affiliations:** 1 Institute of Chemistry, Slovak Academy of Sciences, Dúbravská cesta 5807/9, 845 38 Bratislava, Slovakia; umair.khan@savba.sk; 2 Institute of Medical Biology, Genetics and Clinical Genetics, Faculty of Medicine, Comenius University, Sasinkova 4, 811 08 Bratislava, Slovakia

**Keywords:** extracellular vesicles, exosomes, glycosylation, glycoproteins, glycomics, glycoengineering, mass spectrometry, lectin microarray, biomarkers, regenerative medicine

## Abstract

Glycosylation is a critical determinant of extracellular vesicle (EV) biology, shaping vesicle biogenesis, stability, biodistribution, cellular recognition, and uptake. Because EV glycans mirror disease-associated remodeling of parental cells, EV glycosylation is emerging as both a rich source of biomarkers and a functional regulator of regenerative signaling. This review highlights how altered EV glycosylation generates disease-specific signatures across major cancers, including lung, hepatocellular, colorectal, bladder, ovarian, pancreatic, and prostate cancer, and also discusses evidence in neurological, neuropsychiatric, metabolic, autoimmune, urinary, and musculoskeletal disorders. Beyond diagnostics, we examine the growing role of EV glycosylation in regenerative medicine, where glycan-dependent targeting and tissue interactions contribute to neural, cardiac, renal, skeletal, joint, and skin repair. We further provide an integrated overview of analytical strategies for EV glycosylation research, spanning mass spectrometry-based glycomics and glycoproteomics, affinity-based profiling, lectin microarrays, imaging, spectroscopic methods, advanced biosensing and nanotechnology-based approaches, and emerging artificial intelligence and bioinformatics tools. Current methodological challenges, biosafety issues, translational barriers, and future technologies are also critically discussed. Altogether, this review positions EV glycosylation as a promising interface between EV biology, precision diagnostics, and next-generation regenerative therapeutics.

## 1. Introduction

Over the past decade, extracellular vesicles (EVs), particularly exosomes, have attracted considerable attention in regenerative medicine, biomarker discovery, and tissue engineering due to their role in intercellular signalling and phenotypic modulation [1,2,3]. EVs are lipid bilayer-enclosed nanoparticles that carry proteins, lipids, and nucleic acids and can reprogram recipient cells locally and at distant sites. Rather than acting merely as passive carriers, EVs mediate signal transduction processes that regulate homeostasis as well as pathological responses. Disease states frequently alter EV secretion dynamics. For example, tumour cells often exhibit increased EV release due to elevated metabolic and secretory activity. However, pathological conditions do not universally increase EV abundance, as they more consistently modify EV molecular composition and subtype distribution. Owing to their bioactive cargo, EVs function as cell-free therapeutic mediators by modulating inflammation, promoting angiogenesis, preventing apoptosis, and stimulating tissue repair and stem-cell-like regenerative responses. These properties make EVs promising candidates for biomarkers as well as regenerative and tissue-engineering applications [2,4].

## 2. Origin, Classification and Sources of EVs

**Figure 1:**
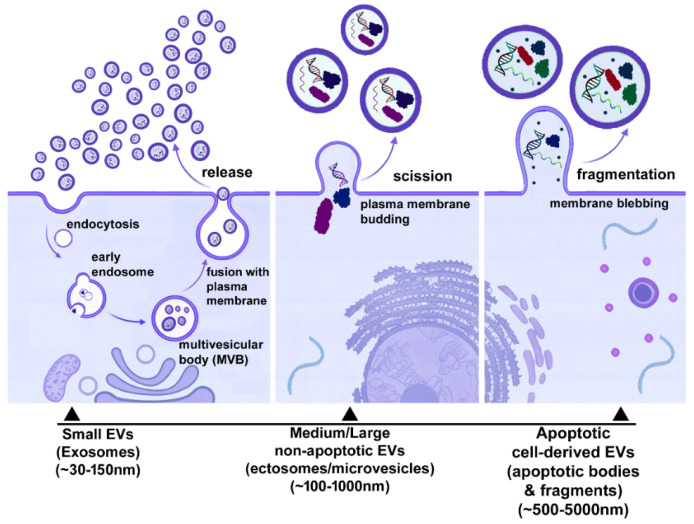
EVs biogenesis. Small EVs originate from the endosomal pathway and are released after multivesicular body fusion with the plasma membrane. Medium/large non-apoptotic EVs form by plasma membrane budding and scission, whereas apoptotic cell-derived EVs arise from membrane blebbing and fragmentation during programmed cell disassembly.

The extracellular space contains a heterogeneous population of lipid bilayer-enclosed particles collectively termed EVs, released by virtually all cell types. EVs act as mediators of intercellular communication in physiological and pathological processes [5]. Historically, EVs were categorised as exosomes, microvesicles, and apoptotic bodies based on presumed biogenesis. However, due to overlapping size ranges and lack of definitive markers, current guidelines recommend classifying EVs using operational terms such as size (e.g., small EVs < 100–200 nm and medium/large EVs > 100–200 nm depending on isolation method), density, molecular composition, or cell of origin. EVs arise from several mechanistically distinct biogenetic pathways, including endosomal trafficking, plasma membrane budding, and apoptosis-associated membrane fragmentation [1,2,3]. Small EVs enriched in endosomal origin (often referred to as exosomes) typically range from ~30–150 nm and are generated within the endosomal system. The endosomal pathway is initiated by endocytosis of the plasma membrane, followed by inward budding of endosomal membranes to produce intraluminal vesicles within multivesicular bodies (MVBs), which are subsequently released into the extracellular space upon MVB fusion with the plasma membrane (exocytosis) [2]. Owing to endosomal sorting mechanisms, small EVs are enriched in specific membrane proteins (e.g., tetraspanins), selected cytosolic components, nucleic acids, and lipids associated with endosomal microdomains. In contrast, medium and large EVs are primarily produced by outward budding and fission of the plasma membrane and may exceed several hundred nanometers in diameter [6]. These vesicles include non-apoptotic ectosomes (historically termed microvesicles) as well as apoptotic cell-derived EVs (Figure 1). They more closely reflect the topology and composition of the parental cell membrane and adjacent cytoplasm and can be released during cell activation, mechanical stress, or apoptosis. Apoptotic cell-derived EVs represent a heterogeneous subset of large EVs generated during programmed cell disassembly by membrane blebbing and fragmentation [7].

Although no marker is entirely specific, small EVs are commonly enriched in tetraspanins (CD9, CD63, CD81) and ESCRT-associated proteins (ALIX, TSG101), while medium/large EVs more frequently display plasma membrane-derived molecules such as integrins and phosphatidylserine [1,8].

**Figure 2:**
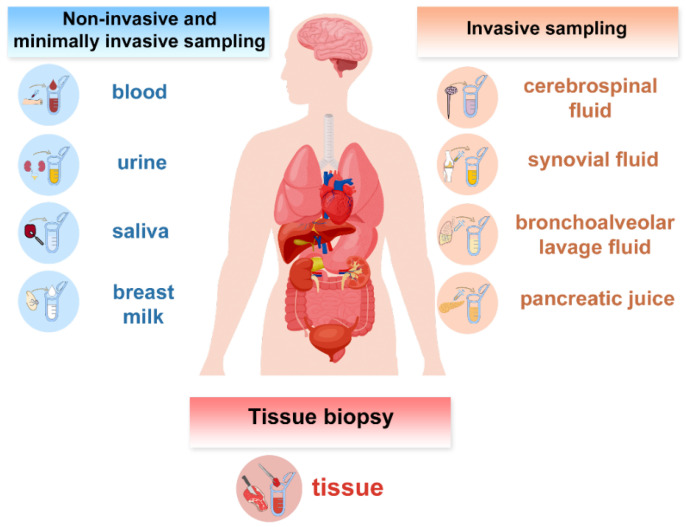
Major human sources of EVs, classified by sampling invasiveness.

EVs can be isolated from multiple biofluids obtained through non-invasive collection (e.g., urine, saliva, breast milk, tears, sweat, semen, cervicovaginal secretions, and sputum) and minimally invasive procedures (e.g., blood collection). These biofluids represent widely used liquid biopsy sources. More invasive sampling procedures provide additional proximal or site-specific EV sources, including cerebrospinal fluid (lumbar puncture), synovial fluid (arthrocentesis), bronchoalveolar lavage fluid (bronchoscopy), bile, pancreatic juice, amniotic fluid, and pathological effusions such as pleural effusions and ascites. Proximal biofluids originate from anatomical sites adjacent to the pathological tissue and therefore often contain higher concentrations of disease-specific extracellular vesicles compared to circulating fluids. Moreover, EVs can also be isolated from tissue-derived materials obtained invasively, such as interstitial fluid from surgical specimens and organ perfusates (Figure 2).

## 3. Glycosylation and Its Function in the EVs

The glycome of EVs provides a functional interface between cellular state and intercellular communication. As a consequence, EV glycosylation simultaneously governs vesicle biodistribution and uptake while offering a highly sensitive molecular fingerprint for diagnostic and therapeutic applications.

Glycosylation is a post-translational modification in which oligosaccharides are covalently attached to proteins or lipids, forming glycoproteins and glycolipids that regulate molecular stability, trafficking, and receptor interactions. In extracellular vesicles (EVs), glycosylation represents a characteristic surface feature largely inherited from the biosynthetic machinery of the parental cell, although limited remodelling may occur after vesicle release [9,10].

EV surface glycans critically regulate vesicle biodistribution, cellular recognition, and uptake. Specific glycan-receptor interactions mediate targeting of EVs to recipient cells. For instance, sialylated glycans interact with Siglec receptors on immune cells, often leading to immunomodulatory or tolerogenic signalling. More broadly, EV glycans serve as ligands for lectins and other glycan-binding receptors, thereby determining cell-type specificity of vesicle internalisation [11,12,13,14].

Glycosylation also contributes to EV stability in the extracellular matrix by limiting aggregation, proteolytic degradation, and immune clearance, thereby prolonging circulation time [15]. In addition, glycan structures participate in cargo sorting during EV biogenesis, influencing the loading efficiency and functional activity of proteins and miRNAs [16,17]. The cellular glycosylation may influence both the total and surface glycosylation of EVs during their biogenesis and release into biofluids, shaping interactions that impact cancer development [18].

Alterations in cellular glycosylation are mirrored in EV glycan signatures, making EV glycome profiling a promising source of diagnostic biomarkers [19]. Similar glycan-dependent recognition mechanisms have been observed in host–pathogen interactions, where parasite-derived EVs exploit host glycan receptors to promote uptake by immune cells [20].

**Figure 3:**
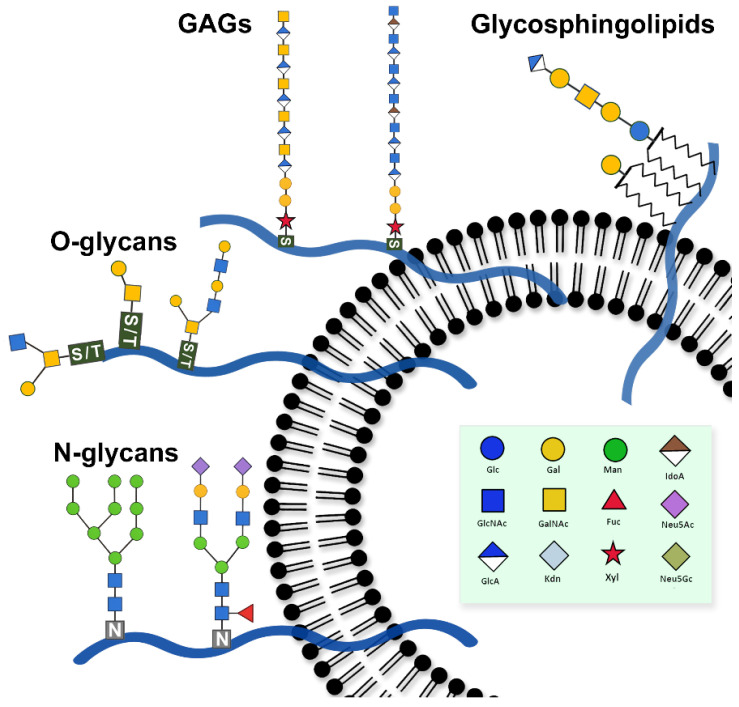
Major classes of glycans present on human extracellular vesicles (EVs), including N-glycans, O-glycans, glycosaminoglycans (GAGs), and glycosphingolipids. Bottom right: schematic representations of monosaccharide building blocks found in human glycans, including Glc (glucose), GlcNAc (N-acetylglucosamine), GlcA (glucuronic acid), Gal (galactose), GalNAc (N-acetylgalactosamine), Kdn (2-keto-3-deoxy-D-glycero-D-galacto-nononic acid), Man (mannose), Fuc (fucose), Xyl (xylose), IdoA (iduronic acid), Neu5Ac (N-acetylneuraminic acid), and Neu5Gc (N-glycolylneuraminic acid). Note: Neu5Gc is not synthesised in humans but may be incorporated into human glycans from dietary sources.

In human cells, glycans are mainly present as N- and O-linked structures on glycoproteins, glycosaminoglycan (GAG) chains in proteoglycans, and glycan moieties of glycolipids such as glycosphingolipids (Figure 3), all of which may contribute to the glycan landscape of EVs, with compositions varying across cell types and physiological states.

Changes in branching, fucosylation, or sialylation substantially modify immune recognition and inflammatory signalling, emphasising the role of EV glycosylation in immune communication and regenerative processes [13,21]. Collectively, EV surface glycans function as a molecular “address code” directing vesicle tropism and intercellular communication.

## 4. EV Glycosylation as a Potential Biomarker

EVs are released by most cell types and carry proteins, lipids, nucleic acids, and glycoconjugates that reflect the physiological and pathological state of their cells of origin. Because EV membranes are derived from defined intracellular compartments, their surface glycan composition is tightly regulated rather than random and mirrors disease-associated alterations in cellular glycosylation. EV glycosylation, therefore, represents a unique biomarker modality that directly captures post-translational regulation of cellular phenotype in a vesicle-specific context and bridges intracellular processes with accessible biofluids.

Glycan structures participate in cell recognition, immune modulation, and tissue remodelling, processes that are frequently dysregulated in disease. Alterations in glycan branching, fucosylation, and sialylation are strongly associated with tumour progression, immune evasion, and metastasis. These changes are reflected in EV surface composition and cargo, enabling minimally invasive detection via liquid biopsy approaches.

Several studies have demonstrated disease-specific EV glycosignatures. For example, urinary EV N-glycome profiling in bladder cancer revealed decreased fucosylation and increased sialylation compared with healthy controls, and a diagnostic model based on selected glycans achieved high discriminatory performance in validation cohorts. These findings highlight the potential of EV glycome profiling as a complementary diagnostic modality, particularly in clinical settings where conventional biomarkers lack sufficient sensitivity or specificity [22].

Importantly, EV glycome profiles do not necessarily replicate the parental cell glycome. Instead, selective sorting during vesicle biogenesis leads to distinct EV-specific glycosignatures, indicating that EV glycosylation encodes an additional regulatory layer of biological information. This divergence may enhance the ability to distinguish EV subpopulations and improve composite biomarker panels capable of capturing disease heterogeneity [23].

Despite this promise, clinical translation remains limited by insufficient standardisation of EV isolation, glycan analysis, and data interpretation. Future progress will depend on advances in high-throughput glycomics, robust analytical pipelines, and integration with multi-omics approaches, enabling the development of clinically translatable EV glycosylation-based biomarkers for precision medicine.

In addition to direct glycan-based signatures, EV-associated glycoproteins have been proposed as complementary biomarkers. Although these studies do not resolve glycan structures, they support the biological and diagnostic relevance of glycosylated EV cargo.

The following sections summarise current evidence across major disease categories in which EV glycosylation and EV-associated glycoproteins have shown diagnostic or pathophysiological relevance, including cancer, neurological and neuropsychiatric disorders, metabolic diseases, and musculoskeletal conditions. Importantly, the strength of evidence is not uniform across these fields. Oncology currently provides the most direct glycan-level evidence, including released glycome, intact glycopeptide, and lectin-based studies, whereas several non-oncological conditions remain supported mainly by indirect glycoprotein, EV cargo, or biologically plausible mechanistic observations. Accordingly, the following sections distinguish, where possible, between established glycosylation-specific findings and emerging areas in which EV glycosylation remains a hypothesis-generating framework. The diseases discussed below share a common feature, pronounced remodelling of cellular glycosylation pathways, reflected in EV glycan signatures detectable in biofluids.

### 4.1. Lung Cancer

Lung cancer comprises two major histological subtypes, small-cell lung cancer (SCLC) and non-small-cell lung cancer (NSCLC), which differ substantially in their molecular characteristics. These differences are also reflected in the glycosylation patterns of small extracellular vesicles (sEVs). Kondo et al. demonstrated that the N-glycan composition of sEVs derived from SCLC and NSCLC cells exhibits distinct and subtype-specific profiles. SCLC-derived sEVs are enriched in glycan motifs characteristic of the brain N-glycome, including bisecting GlcNAc, LacdiNAc, and antennal fucosylation, whereas NSCLC-derived sEVs predominantly contain lung-type core α1,6-fucosylated biantennary and triantennary N-glycans. In addition, the epithelial-associated integrin α6β4 heterodimer was selectively detected on NSCLC-derived sEVs, with its N-glycan profile confirming subtype-specific glycosylation patterns [24].

Complementary evidence highlights the diagnostic relevance of EV-associated glycoproteins. Mucin-1 (MUC1), a heavily glycosylated membrane protein, is enriched in NSCLC-derived exosomes and elevated in patient plasma EVs [25]. Similarly, alpha-2-HS-glycoprotein (AHSG) and extracellular matrix protein-1 (ECM1), combined with carcinoembryonic antigen (CEA), improve diagnostic performance, including in early-stage disease [26].

Recent advances in intact glycoproteomics further extend these observations. Zheng et al. developed a PMs-Ti^4+^-based affinity platform enabling plasma EV capture, in situ tryptic digestion, and intact N-glycopeptide profiling. Using this workflow, the authors identified significant alterations in both EV protein expression and glycosylation in lung cancer patients, including enrichment of sialylated-fucosylated N-glycans and increased glycan heterogeneity at individual glycosylation sites [27]. These findings demonstrate that intact EV glycopeptide profiling can simultaneously resolve disease-associated alterations in both protein and glycan features, thereby extending earlier approaches focused primarily on released glycans or deglycosylated peptides.

More broadly, EV proteomic profiling has identified additional diagnostic and prognostic signatures, including CD155-enriched EV subpopulations and multi-protein panels that discriminate NSCLC patients from controls and stratify survival risk [28,29].

Taken together, these findings indicate that EV glycosylation and glycoprotein signatures may support subtype discrimination and clinically relevant biomarker development in lung cancer.

### 4.2. Hepatocellular Cancer (HCC)

Urinary EVs (uEVs) have been profiled at the intact N-glycopeptide level to identify glycosylation-based biomarkers for hepatocellular carcinoma (HCC). A comprehensive glycoproteomic study identified 756 intact N-glycopeptides corresponding to 154 glycosites on 107 glycoproteins, with 344 glycopeptides differentially expressed between HCC patients and controls. Site-specific glycosylation changes were observed on proteins such as galectin-3-binding protein (LGALS3BP), polymeric immunoglobulin receptor (PIGR), and kininogen-1 (KNG1), while ASPP2-associated glycopeptides were reduced [30]. These findings demonstrate that uEV glycoproteomics captures disease-specific, site-resolved glycosylation alterations and represents a promising non-invasive biomarker source for HCC detection. HCC thus represents a clinically relevant example of site-specific EV glycosylation changes with direct diagnostic potential.

### 4.3. Colorectal Cancer (CRC)

CRC is characterised by extensive remodelling of cellular glycosylation, which is reflected in extracellular vesicles (EVs). Lectin microarray profiling revealed subtype-specific EV glycan signatures, with Ulex europaeus agglutinin I (UEA-I), a fucose-binding lectin, showing selective binding to a CRC subtype. These patterns correlate with glycosylation-related gene expression and enable dynamic monitoring of tumour progression [31].

Glycan-dependent EV subpopulation analysis further enhances diagnostic sensitivity. Increased co-expression of CD151 and CD63 on EVs, combined with lectin-based assays (e.g., CEA–ConA, CA125–WGA), improves early CRC detection compared with conventional biomarkers alone [32].

Proteomic studies provide complementary evidence, identifying EV-associated glycoproteins such as fibrinogen beta chain (FGB), β2-glycoprotein 1, and alpha-1-acid glycoprotein 1 (ORM1) as diagnostic and prognostic markers [33,34].

Thus, CRC represents a comparatively well-supported model in which EV glycosylation and glycoprotein signatures jointly provide diagnostically relevant information. However, further validation using harmonised EV isolation and glycan-analysis workflows will be required before these signatures can be considered clinically mature.

### 4.4. Pancreatic Cancer

Pancreatic cancer lacks reliable biomarkers for early detection, contributing to poor prognosis. EV glycosylation has emerged as a promising minimally invasive approach. Lectin microarray profiling identified increased O-glycan–associated EV subpopulations in patient serum, particularly those recognised by Agaricus bisporus agglutinin (ABA) and Amaranthus caudatus agglutinin (ACA) [35]. These glycan-defined EV populations were elevated even in patients negative for the conventional biomarker CA19-9, and decreased following tumour resection, supporting their potential for both diagnosis and disease monitoring. Although still requiring validation, pancreatic cancer underscores the clinical potential of EV O-glycan signatures, particularly in biomarker-negative patient subgroups. At present, however, the evidence remains exploratory and should be interpreted as an indication of biomarker promise rather than proof of clinical readiness.

### 4.5. Bladder Cancer

Bladder cancer represents one of the most well-established examples of the biomarker potential of extracellular vesicle (EV) glycosylation, particularly due to the direct contact between tumour tissue and urine. Comprehensive N-glycome profiling of urinary EVs from a large cohort revealed extensive glycan diversity, with 252 N-glycan structures identified and significant differences between patients with bladder cancer and healthy controls. Notably, urinary EVs from bladder cancer patients exhibited decreased fucosylation and increased sialylation, indicating disease-associated remodelling of glycan composition. In addition to compositional changes, alterations in glycan diversity and site-specific glycosylation patterns were observed, supporting the concept that EV glycosylation reflects selective cargo sorting rather than passive shedding from parental cells. Functional analyses further suggested that EVs derived from bladder cancer cells may influence recipient cell behaviour, including processes associated with epithelial–mesenchymal transition and increased invasiveness, highlighting the biological relevance of EV glycosylation changes. Importantly, a diagnostic model based on a panel of eight N-glycans demonstrated strong discriminatory performance, with AUC values of 0.88 and 0.86 in independent validation cohorts, supporting the potential of urinary EV glycome profiling as a non-invasive diagnostic approach. These results pave the way for developing EV-based glycan assays for clinical bladder cancer screening and also offer insights into glycosylation studies for other cancers [22].

Bladder cancer, therefore, represents a benchmark disease in which urinary EV glycosylation shows both strong diagnostic performance and biological relevance, supporting its use as a robust source for non-invasive biomarker development. These findings are consistent with broader evidence highlighting urinary EVs as a promising platform for bladder cancer liquid biopsy, although clinical implementation will still require assay standardisation and prospective validation across independent cohorts [36].

### 4.6. Ovarian Cancer

Ovarian carcinoma cells release extracellular vesicles with glycosylation patterns that differ from those of total cellular membranes, supporting the concept that EV-associated glycans represent selectively enriched molecular signatures rather than passive reflections of the parental cell surface. Early glycomic and glycoproteomic studies showed that ovarian cancer-derived exosomes are enriched in complex N-glycans containing α2,3-linked sialic acid, fucosylation, bisecting GlcNAc, and LacdiNAc motifs, as well as O-glycans carrying the T-antigen. LGALS3BP has been identified as a key EV glycoprotein carrying complex sialylated N-glycans [37,38].

More recent work has provided stronger mechanistic evidence for disease-associated EV glycosylation in high-grade serous ovarian carcinoma (HGSOC). EVs derived from HGSOC displayed distinct glycosignatures relative to non-cancer controls, including reduced overall N-glycosylation and increased α2,6-sialylation, particularly in medium/large EVs. These changes were associated with altered expression of glycosylation machinery in tumour tissue, including increased ST6GAL1 and reduced ST3GAL3/4 expression, indicating that EV glycan profiles reflect underlying glycosylation remodelling in ovarian cancer [39]. Complementary proteomic studies identified MUC1-containing EV panels with strong performance for early-stage detection [40]. Ovarian cancer, therefore, represents one of the strongest and most mechanistically supported examples of EV glycosylation-based biomarkers.

### 4.7. Prostate Cancer (PCa)

PCa is associated with altered extracellular vesicle composition, including changes in glycans, glycolipids, and glycoprotein cargo. Direct evidence for the biomarker relevance of EV-associated glycosylation comes from urinary studies showing that PCa is associated with increased release of urinary extracellular vesicles together with an altered urinary N-glycosylation profile, supporting the concept that EV-related glycan changes can be detected non-invasively in this disease [41].

Additional mechanistic studies support the biological relevance of EV glycosylation in PCa. Exosomes released by the PC-3 prostate cancer cell line were shown to be markedly enriched in glycosphingolipids, as well as cholesterol, sphingomyelin, and phosphatidylserine, indicating selective lipid and glycolipid sorting into PCa-derived EVs [42]. Furthermore, increased expression of the core fucosyltransferase FUT8 in PCa cell models reduced EV secretion and altered the composition of EV-associated glycoproteins, linking aberrant fucosylation to EV biogenesis and cargo remodeling in PCa [43].

In addition to direct glycan-related evidence, urinary EV proteomics has identified disease-associated protein signatures across Gleason score groups, supporting the broader biomarker relevance of urinary EVs in PCa [44]. However, these studies do not directly resolve glycan structures and should therefore be interpreted as complementary to, rather than substitutes for, EV glycosylation-based biomarkers. Thus, PCa represents a disease where mechanistic links between glycosylation and EV biology are strong, but clinical glycan biomarker evidence is still emerging.

### 4.8. Renal Cancer

Compared with other malignancies, direct evidence for EV glycosylation biomarkers in renal cancer remains limited. Most studies focus on EV-associated proteins and nucleic acids.

Tissue-derived EVs (Ti-EVs) and corresponding uEVs analyses have identified subtype-specific biomarker panels with high diagnostic performance (AUC > 0.9) in clear renal cell carcinoma (RCC) subtypes [45]. EV-derived microRNAs and circular RNAs further provide prognostic information [46].

Although glycan structures are not directly resolved, these findings indicate that EV cargo reflects disease-associated renal cell states. Whether these signatures are directly linked to altered EV glycosylation remains to be determined. Renal cancer highlights a clear gap, where EV-based biomarkers are established but glycosylation-specific studies remain underdeveloped.

### 4.9. Urinary Non-Cancerous Diseases

uEVs represent a promising non-invasive source of biomarkers also in kidney diseases beyond cancer. uEVs are released by epithelial and other cell types along the nephron and urogenital tract and carry proteins, nucleic acids, lipids, and glycoconjugates that reflect the physiological and pathological state of their cells of origin [47,48].

Direct evidence for disease-associated EV glycosylation in non-cancerous kidney diseases has been demonstrated using lectin-based glycomic approaches. In a pilot study, lectin microarray profiling of uEVs from patients with autosomal dominant polycystic kidney disease (ADPKD) revealed distinct glycan signatures compared with healthy controls. Differences in binding intensity were observed for 6 out of 43 lectins, and multivariate analysis enabled clear separation between disease and control groups. Importantly, these glycan profiles were shown to be distinct from co-isolated urinary proteins, supporting the specificity of EV-associated glycosylation patterns [49]. These findings indicate that uEV glycosylation reflects disease-associated alterations in kidney function and may serve as a source of non-invasive biomarkers in chronic kidney diseases. However, current evidence remains limited to small exploratory studies, and further validation across different kidney disease etiologies is required.

### 4.10. Neurological and Neuropsychiatric Disorders

EVs have enabled minimally invasive access to central nervous system (CNS) pathology because neurons, astrocytes, microglia, and other neural cell types release them, and their molecular cargo can enter peripheral biofluids. Accordingly, EVs have emerged as promising liquid biopsy candidates for neurological and neuropsychiatric disorders, including Alzheimer’s disease (AD), Parkinson’s disease (PD), and related conditions in which antemortem diagnosis remains challenging. However, most currently studied EV biomarkers in these diseases are proteins, lipids, or nucleic acids rather than direct glycosylation-based signatures [50,51,52].

Direct evidence for EV glycosylation as a biomarker in CNS-related disorders has recently been demonstrated in childhood epilepsy. Serum-derived EV N-glycome profiling identified 47 characteristic N-glycans that distinguished children with epilepsy from healthy controls and further differentiated focal from generalized epilepsy using a two-step machine-learning model. Importantly, EV-specific glycan signatures showed superior diagnostic performance compared with serum-derived N-glycans, supporting the concept that EV glycosylation provides disease-specific and biologically enriched biosignatures [53].

Additional evidence for the biomarker relevance of EV glycosylation has emerged in neuropsychiatric disease. Altered glycosylation of von Willebrand factor (vWF) associated with circulating EVs was shown to predict depression, indicating that EV-associated glycosylation changes may extend beyond neurodegeneration and may serve as biomarkers across a broader spectrum of CNS-related disorders [54].

By contrast, in major neurodegenerative diseases such as AD and PD, EV biomarker research remains dominated by protein-based EV markers (tau, amyloid-β, α-synuclein). Although these findings strongly support EV-based liquid biopsy approaches, they do not yet establish EV glycosylation as a mature biomarker class in these disorders [55,56].

Current evidence indicates that EV glycosylation-based biomarkers in neurological and neuropsychiatric disorders are emerging but remain less developed than protein- or RNA-based EV biomarkers. These findings also suggest that EV glycosylation may capture disease-relevant molecular changes not detectable by bulk serum glycomics. Epilepsy and depression currently provide the most direct evidence of clinically relevant EV glycan signatures, but these observations require replication in larger, disease-specific cohorts and comparison with established CNS-derived EV biomarker platforms. This represents a promising but still early-stage field that warrants further systematic investigation.

### 4.11. Type 2 Diabetes Mellitus (T2DM) and Related Metabolic Disorders

Compared with several malignancies, direct evidence for EV glycosylation biomarkers in T2DM is limited. Most studies focus on EV proteins, miRNAs, and cellular origin. Nevertheless, EV-mediated interorgan communication is highly relevant to metabolic disease, and altered EV uptake and biodistribution may plausibly be influenced by glycosylation-dependent mechanisms [57].

Current biomarker evidence in T2DM is therefore dominated by non-glycan EV signatures. Proteomic and transcriptomic EV biomarkers have been identified in diabetic complications, including retinopathy and systemic insulin resistance [58]. Circulating EV phenotyping in T2DM has also revealed altered large EV subpopulations supporting a link between EVs, inflammation, and vascular stress [59,60].

Although these studies do not directly resolve EV glycan structures, they indicate that EV cargo reflects disease-associated metabolic remodelling and may be shaped by upstream cellular processes, including glycosylation. At present, however, EV glycosylation cannot yet be considered a mature biomarker class in T2DM. Therefore, any functional or diagnostic role of EV glycosylation in metabolic disease should currently be regarded as biologically plausible but not yet directly established. This represents an important gap in the field, particularly given the central role of glycosylation abnormalities in diabetes biology and the growing relevance of EV-based liquid biopsy in metabolic disease [57].

### 4.12. Musculoskeletal and Joint Diseases

EVs are key mediators of intercellular communication in musculoskeletal and joint diseases, such as osteoarthritis (OA), rheumatoid arthritis (RA), and intervertebral disc degeneration (IVDD). EVs released from synoviocytes, chondrocytes, and immune cells are present in synovial fluid and circulation and reflect local inflammatory and degenerative processes, making them attractive candidates for minimally invasive biomarker discovery [61].

Compared with oncology, direct evidence for EV glycosylation as a biomarker in musculoskeletal diseases remains limited. Most studies have focused on EV-associated proteins, miRNAs, and inflammatory markers rather than on detailed characterisation of EV glycan structures. Nevertheless, EV membranes are enriched in glycoproteins, including tetraspanins such as CD9, CD63, and CD81, whose expression levels correlate with disease activity in OA and RA, suggesting that glycosylated EV surface components may carry diagnostic information [62,63]. In RA, EV-associated proteins and immune complexes reflect systemic inflammation and correlate with clinical disease activity markers (DAS28, CRP, ESR). Although these studies primarily assess protein abundance, many of the identified EV proteins are glycosylated and participate in immune regulation, supporting a potential role of EV glycosylation in disease-relevant signalling [64,65]. Similarly, in OA, synovial fluid-derived EVs show disease-stage-dependent changes in abundance, size distribution, and molecular composition. Proteomic analyses indicate enrichment of extracellular matrix (ECM)-related proteins, many of which are glycoproteins involved in cell adhesion and matrix remodeling, suggesting that EV-associated glycoproteins may reflect early degenerative changes in joint tissues [66]. In IVDD, EV proteomic profiling has revealed stage-specific differences in ECM-associated proteins, including glycosylated molecules involved in matrix organisation and cell–matrix interactions [67]. Beyond OA and RA, EV biomarkers have also been investigated in other autoimmune and inflammatory rheumatic diseases, including psoriatic arthritis, systemic lupus erythematosus, and spondyloarthritides. However, direct EV glycosylation-specific evidence remains scarce [68].

Overall, autoimmune musculoskeletal diseases illustrate a significant translational gap, where EV glycosylation remains largely unexplored despite strong biological rationale. This is particularly notable in diseases such as systemic lupus erythematosus, where aberrant glycosylation of circulating immunoglobulins is well established, yet corresponding EV glycosylation signatures remain largely unexplored. Accordingly, current evidence in musculoskeletal and rheumatic diseases should be viewed primarily as indirect support for future EV glycosylation studies rather than as established biomarker evidence. Direct glycomic and glycoproteomic analyses of disease-relevant EV populations from synovial fluid, plasma, and tissue-proximal compartments will be needed to define whether EV glycosylation provides information beyond conventional inflammatory and proteomic markers.

### 4.13. Integrative Perspective on EV Glycosylation Biomarkers

EV glycosylation is emerging as a biologically informative and clinically promising biomarker class that links intracellular glycosylation remodelling with accessible biofluids. Across the disease categories discussed here, EV-associated glycans and glycoproteins reflect disease-specific alterations in cellular glycosylation pathways. However, the maturity of evidence differs substantially between disease areas. The strongest evidence currently comes from oncology, where EV glycome profiling, particularly in bladder, ovarian, colorectal, and pancreatic cancers, has demonstrated robust diagnostic potential, including high discriminatory performance and disease-specific glycosignatures. By contrast, in non-oncological conditions such as neurological, neuropsychiatric, metabolic, and autoimmune diseases, EV glycosylation-based biomarkers remain less developed, with most studies still focused on proteins and nucleic acids and only limited direct glycan-level analyses. Nevertheless, emerging findings in epilepsy and selected neuropsychiatric conditions suggest that EV glycosylation may reveal molecular signatures not captured by conventional serum glycomics. Across disease settings, a recurring theme is the selective sorting of glycosylated cargo into EVs, indicating that EV glycosylation conveys biological information beyond the parental cell glycome. This concept is well supported in several cancer models but remains less directly demonstrated in many non-oncological settings. Despite this promise, the field remains constrained by limited standardisation of EV isolation and glycomic workflows, as well as by the scarcity of large-scale validation studies. Table 1 summarises disease-associated changes in EV glycosylation discussed in this review in those conditions for which sufficiently robust glycan- or glycoprotein-level evidence is currently available, together with their level of evidence and biomarker potential. Future progress will depend on integrating glycomics with proteomics and transcriptomics within multi-omics frameworks, thereby enabling the development of robust, clinically translatable EV glycosylation-based biomarkers for precision medicine.

## 5. Functional Role of EV Glycosylation in Regenerative Medicine

Beyond biomarker discovery, EV glycosylation may also contribute to functional processes relevant to tissue repair and regeneration. Glycan-dependent mechanisms that shape EV glycosignatures also regulate vesicle targeting, biodistribution, and interaction with recipient cells. EV glycosylation, therefore, represents both a biomarker of disease state and a determinant of EV-mediated biological activity, providing a direct conceptual link to regenerative applications. However, in regenerative medicine, direct glycome-level evidence remains less extensive than in oncology, and many proposed roles of EV glycosylation are currently inferred from glycoengineering, biodistribution, receptor-binding, or EV-uptake studies rather than from systematic glycomic profiling of therapeutic EVs.

EVs, particularly exosomes, have emerged as promising therapeutic agents in regenerative medicine due to their ability to mediate intercellular communication and deliver bioactive cargo, including proteins, lipids, and nucleic acids. Beyond these cargo-mediated effects, EV surface glycosylation is increasingly recognised as a potential layer of functional regulation, influencing biodistribution, cellular targeting, uptake, and ultimately therapeutic efficacy. Glycosylation enables EVs to interact with glycan-binding receptors such as lectins and Siglecs on recipient cells, thereby directing vesicle tropism toward specific tissues and cell types. These glycan–receptor interactions are particularly relevant in regenerative settings, where efficient and selective delivery to injured tissues is essential. Nevertheless, the extent to which endogenous, non-engineered EV glycosylation determines regenerative outcomes remains incompletely defined and likely varies across tissue contexts. In addition, EV glycosylation contributes to circulation stability by reducing immune recognition and enzymatic degradation, thereby prolonging vesicle half-life in vivo. Emerging evidence further suggests that glycosylation influences EV biogenesis and cargo sorting, contributing to functional heterogeneity and tissue-specific effects.

From a translational perspective, EV glycosylation represents a tractable target for bioengineering strategies. Modification of surface glycans has been shown to enhance tissue targeting, biodistribution, and therapeutic performance in preclinical models, highlighting glycosylation as a tunable parameter in EV-based therapies [69,70].

To reflect both tissue-specific applications and shared regenerative mechanisms, the following sections are organised into organ-level repair contexts and cross-cutting regenerative processes, particularly angiogenesis and immunomodulation, in which EV glycosylation plays a common functional role.

### 5.1. Neural Regeneration

Neural repair requires coordinated communication between neurons, glial cells, and vascular components. EV-based therapies have demonstrated regenerative potential in stroke, traumatic brain injury, and neurodegeneration, primarily through delivery of neuroprotective cargo [71,72]. However, these studies largely reflect general EV-mediated mechanisms.

EV glycosylation may contribute to interactions with neural cells and influence delivery across the blood–brain barrier. Glycan–receptor interactions, including binding to lectins and sialic acid–recognising receptors, have been implicated in EV uptake by neurons, astrocytes, and microglia, indicating that glycosylation could contribute to cell-type-specific targeting within the central nervous system. In addition, EV glycosylation may influence the ability of vesicles to cross the blood–brain barrier (BBB), a key requirement for therapeutic application in neurological diseases. Surface glycans, particularly sialylated structures, have been associated with enhanced circulation stability and reduced immune clearance, thereby increasing the likelihood of EV delivery to neural tissues [70]. However, direct evidence linking defined EV glycan structures to functional neural regeneration remains limited.

From a translational perspective, glycoengineering approaches have been explored to enhance EV targeting to neural tissues. Modulation of EV surface glycans, including enzymatic desialylation or introduction of specific glycan motifs, has been shown to alter EV uptake efficiency and biodistribution in preclinical models, supporting the concept that EV glycosylation can be actively manipulated to improve regenerative outcomes [69]. Neural regeneration is an emerging field in which EV glycosylation may influence targeting and biodistribution, although its precise functional contribution to neural repair remains incompletely defined.

### 5.2. Myocardial Regeneration

Myocardial repair following ischemic injury requires coordinated cardiomyocyte survival, angiogenesis, and immune modulation. EVs derived from mesenchymal stromal cells (MSC) and cardiosphere-derived cells promote these processes through delivery of bioactive cargo [73]. EV surface glycans are likely to influence interactions with cardiomyocytes, endothelial cells, and immune cells, thereby regulating tissue-specific targeting. Glycosylation also affects circulation dynamics, enhancing EV persistence and increasing delivery efficiency to ischemic myocardium [70]. Glycoengineering approaches further demonstrate improved cardiac targeting and therapeutic efficacy [69]. These approaches highlight the potential of glycosylation as a tunable parameter for optimising EV-based cardiac therapies. Thus, myocardial regeneration represents a translational context in which glycosylation may contribute to EV targeting and biodistribution, although direct glycomic characterisation and causal evidence in cardiac repair models remain limited. These processes are closely linked to vascular regeneration, where EV glycosylation has more direct experimental support in endothelial targeting (see Section 5.8).

### 5.3. Renal Regeneration

Renal regeneration relies on interactions between tubular epithelial cells, immune cells, and endothelial cells within the vascular compartment. EVs promote tubular survival and reduce inflammation through transfer of EV-associated proteins and RNAs delivered by MSC and other reparative cell populations [74,75,76].

In this setting, EV glycosylation is particularly relevant for tissue targeting. Surface glycans regulate EV biodistribution and uptake in the injured kidney. Direct evidence comes from glycoengineered EVs, where hyaluronic acid-modified vesicles selectively accumulate in injured renal tissue via CD44- and TLR4-mediated interactions, resulting in improved therapeutic efficacy, demonstrating that engineered EV glycosylation can be used to direct regenerative delivery to damaged kidney tissue [77].

These findings indicate that glycosylation can actively determine renal tropism and therapeutic performance in engineered EV systems, positioning kidney regeneration among the most advanced examples of glycan-mediated EV targeting. However, broader conclusions about endogenous EV glycosylation in renal repair will require systematic profiling of native therapeutic EV preparations and validation across injury models.

### 5.4. Liver Regeneration

Liver regeneration involves coordinated interactions among hepatocytes, immune cells, and endothelial compartments. EVs contribute to these processes by delivering regulatory cargo. The liver is uniquely relevant for EV glycosylation because specialised glycan-recognition systems, particularly the asialoglycoprotein receptor (ASGPR), mediate uptake of galactose- and N-acetylgalactosamine-terminated glycans. This receptor plays a central role in the clearance and uptake of glycosylated particles, including EVs, by hepatocytes. Experimental studies show that desialylation of EVs enhances hepatic uptake via ASGPR, demonstrating that glycan composition directly governs liver tropism [69,78]. This mechanism is particularly relevant for regenerative therapies, where efficient delivery of EVs to hepatocytes is essential. In addition to targeting, EV glycosylation may influence interactions with Kupffer cells and hepatic immune responses. Glycan-dependent recognition by hepatic lectins can modulate EV clearance, immune activation, and inflammatory signalling, thereby affecting the regenerative microenvironment. These findings make liver regeneration one of the clearest contexts in which glycoengineering can be used to enhance EV targeting, although systematic glycomic profiling of liver-directed EV therapies remains limited. Therefore, the evidence is strongest for glycan-dependent biodistribution and hepatic uptake, whereas direct links to regenerative efficacy remain less fully established.

### 5.5. Bone and Skeletal Regeneration

Bone regeneration requires coordinated activity of osteoblasts, osteoclasts, and mesenchymal progenitors within a mineralised extracellular matrix. EVs promote osteogenesis primarily through delivery of regulatory cargo [79,80]. EV glycosylation may regulate interactions with bone matrix components, facilitating retention at injury sites and influencing uptake by osteogenic cells. Indirect evidence supports a role for glycosylated EV membrane proteins, such as CD9, in bone repair, as demonstrated by impaired regeneration in CD9-deficient models [81]. Although direct glycan-level evidence remains limited, glycosylation may contribute to EV localisation and cell-specific targeting in bone and skeletal regeneration. At present, however, this remains primarily an inferred mechanism based on the glycoprotein-rich nature of EV membranes and indirect functional evidence rather than direct glycomic analysis.

### 5.6. Cartilage and Joint Regeneration

Cartilage repair occurs within a highly glycosylated extracellular matrix enriched in proteoglycans and GAGs. EVs modulate inflammation and support matrix remodelling in osteoarthritis and rheumatoid arthritis [82,83]. However, most available studies focus on EV cargo and do not directly address the role of EV glycosylation.

In this environment, EV surface glycans are likely to influence retention within cartilage and synovial tissues through glycan-mediated interactions. Glycosylation may also regulate uptake by chondrocytes and synovial cells, as well as immune cell interactions in inflammatory conditions. Indirect evidence supports a role for glycosylated EV membrane proteins in joint biology. Tetraspanins such as CD9, CD63, and CD81, glycosylated proteins commonly enriched on EVs, have been associated with disease activity and immune signalling in rheumatoid arthritis, suggesting that EV surface glycoproteins contribute to cell–cell communication within the joint environment [62]. From a translational standpoint, EV glycosylation represents a potential target for improving joint-specific delivery, particularly in diseases where local retention within synovial or cartilage compartments is critical. However, current evidence remains largely indirect, and direct glycan-resolved studies are needed to determine whether EV glycosylation has a causal role in joint repair or primarily reflects broader changes in EV surface composition.

### 5.7. Skin and Wound Regeneration

Cutaneous wound healing involves dynamic interactions between epithelial, stromal, and immune cells. EVs promote angiogenesis, re-epithelialisation, and modulation of inflammation [84,85]. EV glycosylation contributes to interactions with extracellular matrix components and immune cells, enhancing retention at wound sites and regulating local inflammatory responses. This property is particularly important in wound healing, where spatial localisation of EV activity is a key determinant of therapeutic efficacy. Surface glycoproteins may also interact with coagulation pathways, supporting early wound stabilisation [86]. More direct translational support now comes from glycoengineered EV systems. FUT7-mediated surface glycoengineering has been used to generate EVs with enhanced targeting capacity and improved angiogenesis-promoting activity in diabetic wound models, indicating that controlled modification of EV glycans can strengthen regenerative efficacy in the skin [87]. This provides one of the more direct examples of glycoengineering in regenerative EV therapy, although systematic comparison with native EV glycosylation profiles remains limited.

### 5.8. Angiogenesis and Vascular Regeneration

Angiogenesis is a central component of tissue repair. EVs contribute primarily through pro-angiogenic cargo, but glycosylation also plays a key role in endothelial targeting. Glycan-dependent interactions with endothelial receptors and glycocalyx components promote EV accumulation at sites of vascular injury, enhancing local signalling. This is particularly important in ischemic tissues, where efficient targeting of EVs to damaged vasculature is a key determinant of therapeutic efficacy. Glycoengineering approaches further support this concept: reconfigurable glycocalyx engineering has enabled display of defined glycan ligands, including sialyl Lewis X (sLeX), on EV surfaces, resulting in enhanced cell-specific targeting and providing direct proof that EV glycosylation can be manipulated to improve vascular delivery [69,88]. Thus, vascular regeneration represents a key mechanistic context in which EV glycosylation can influence tissue localisation, highlighting the translational importance of glycoengineering. Nevertheless, most evidence currently derives from engineered EV systems, and further studies are required to define how endogenous EV glycan diversity regulates vascular repair in vivo.

### 5.9. Immunomodulation in Regeneration

Effective regeneration requires controlled immune responses and resolution of inflammation. EV glycosylation regulates immune cell recognition through interactions with lectins and Siglecs, influencing immune activation, tolerance, and clearance [70]. Direct mechanistic evidence for glycan-dependent EV uptake has been demonstrated for Siglec-6, which mediates internalisation of EVs through a noncanonical glycolipid-binding mechanism [89]. This finding is particularly important because it shows that EV-associated glycoconjugates can directly determine immune-cell engagement rather than merely modulate it indirectly. Glycoengineering approaches further support the potential to modulate immune responses by altering EV surface glycans, thereby altering receptor recognition, biodistribution, and persistence. Although direct glycan-resolved evidence remains limited across regenerative systems, immunomodulation remains one of the most mechanistically grounded roles of EV glycosylation. Future work should determine whether specific EV glycan motifs reproducibly promote immune tolerance, inflammatory resolution, or clearance in tissue-repair settings.

### 5.10. Mechanistic and Translational Perspective on EV Glycosylation in Regeneration

Across regenerative contexts, several shared functional principles emerge. EV glycosylation contributes to tissue tropism through interactions with glycan-binding receptors, regulates vesicle retention within extracellular matrices, and modulates circulation stability and immune recognition. These mechanisms collectively influence the spatial distribution and functional persistence of EVs in injured tissues. In addition, glycoengineering studies demonstrate that EV surface glycans can be actively modified to enhance tissue specificity, in vivo distribution, and therapeutic efficacy, highlighting glycosylation as a tunable parameter in EV-based regenerative strategies. However, the strength of evidence varies substantially across tissues. The most direct support currently comes from glycoengineered EV systems and defined receptor-mediated uptake models, whereas many tissue-specific regenerative applications remain supported by indirect evidence from EV biodistribution, glycoprotein cargo, or general EV functional studies. These findings position EV glycosylation as a key functional interface between vesicle biology and tissue repair. Although direct glycome-level evidence remains uneven across tissues, converging mechanistic and translational data support its role as an important determinant of regenerative outcomes and a promising target for optimising EV-based therapies. The regenerative contexts in which EV glycosylation has been directly implicated or experimentally engineered, highlighting glycan-related mechanisms, functional effects, and current evidence level, are summarised in Table 2.

The functional relevance of EV glycosylation in both diagnostics and regeneration underscores the need for precise analytical characterisation. Glycan-dependent mechanisms that govern EV targeting, biodistribution, and biological activity require methodologies capable of resolving glycan structure and functional context. This has driven the development of complementary analytical platforms, including MS-based glycomics, lectin-based assays, and advanced biosensing approaches, which are discussed in the following section.

## 6. Analytical Methods for EV Glycosylation

Analytical investigation of EV glycosylation requires methods capable of addressing both structural heterogeneity and low-abundance analytes. Depending on the analytical objective, EV glycosylation may be studied at the level of released glycans, intact glycopeptides and glycoproteins, or glycolipids. Current workflows, therefore, combine orthogonal strategies, including mass spectrometry-based glycomics and glycoproteomics, lectin-based profiling, affinity enrichment, biosensing, and spectroscopic approaches. Collectively, these methods provide complementary information on glycan composition, site-specific glycosylation, surface accessibility, and functional interactions, and are increasingly being adapted for EV-focused applications [9,13,90,91].

### 6.1. Pre-Analytical Considerations: EV Isolation and Sample Preparation

Pre-analytical handling is a major determinant of EV glycosylation readouts. Because EV preparations frequently co-isolate abundant soluble glycoproteins, lipoproteins, protein aggregates, or urinary polymers such as Tamm–Horsfall protein, the observed glycan profile may reflect both true EV-associated glycans and matrix-derived contaminants. Accordingly, EV isolation strategy must be aligned with the downstream analytical aim, whether this is surface glycan profiling, glycoproteomics, or glycan-targeted capture [9,23].

Recent studies directly show that the isolation method can alter the detectable glyco-properties of EVs. It was demonstrated that additional purification steps after ultracentrifugation changed the ratio of lectin-positive small versus larger EV subpopulations, indicating that size-exclusion chromatography (SEC) and microfiltration can influence the apparent surface glycosylation profile of isolated vesicles [92]. Similarly, it was shown that ultracentrifugation and immunoaffinity-based MagCapture enrichment yield different particle characteristics and different downstream glycoproteomic coverage, with the optimal choice depending on starting volume and study objective [93].

Sample preparation for EV glycan analysis often requires an additional enrichment step because glycoproteins may be low in abundance, and glycans generally ionise poorly. Common enrichment strategies include lectin affinity capture, hydrazide chemistry, boronic acid affinity chromatography (BAC), hydrophilic interaction chromatography (HILIC), and porous graphitised carbon chromatography (PGC) workflows. Importantly, these steps should be considered part of the analytical design rather than neutral processing steps, because each enrichment strategy introduces selectivity toward specific glycoforms or glycan classes [14,24].

Taken together, EV glycosylation analysis is highly sensitive to pre-analytical choices. Isolation, purification, and enrichment steps do not merely improve signal quality; they also shape which EV-associated glycans become analytically visible. This must be considered when comparing studies and when designing clinically translatable workflows.

### 6.2. Analytical Targets: Glycans, Glycoproteins, and Glycolipids

A critical distinction in EV glycoanalytics is the nature of the molecular target. Released glycan analysis interrogates glycans after enzymatic or chemical release from proteins or lipids and provides the broadest view of glycan composition, branching, fucosylation, and sialylation, but usually loses information about the carrier molecule. Glycopeptide and glycoprotein analysis preserves the linkage to the protein backbone and therefore enables site-specific assignment of glycosylation, albeit at the cost of greater analytical complexity. Glycolipid analysis focuses on glycosphingolipids and other membrane-associated glycoconjugates that are particularly relevant for EV membrane organisation, targeting, and cell recognition [13,91].

These analytical levels are complementary rather than interchangeable. Released glycans are most informative for profiling global glycome shifts; glycopeptides are essential when biological interpretation depends on the modified protein and glycosylation site; and glycolipid-oriented approaches are particularly important when EV surface recognition or membrane composition is under investigation. Clarifying the analytical target is therefore a prerequisite for selecting the appropriate methodology and for interpreting the biological meaning of EV glycosylation data.

### 6.3. Mass Spectrometry-Based Glycomics and Glycoproteomics

Mass spectrometry (MS) represents the central analytical platform for characterising EV glycosylation, enabling high-sensitivity and high-resolution analysis of glycan structures, glycopeptides, and glycoproteins. Current MS-based workflows can be broadly divided into released glycan analysis, glycopeptide/glycoprotein analysis, and glycolipid analysis, each providing complementary structural and functional information.

Liquid chromatography coupled to tandem MS (LC–MS/MS), typically using electrospray ionisation (ESI), constitutes the core analytical approach for EV glycomics and glycoproteomics. Chromatographic separation (e.g., HILIC, reversed-phase, or PGC) reduces sample complexity and enables detection of low-abundance glycoforms in complex biofluids, therefore playing a central role in MS-based EV glycoanalytics. These approaches are essential for analysing complex EV samples, where co-isolated proteins and glycoforms would otherwise compromise sensitivity and structural resolution. In EV studies, LC–MS/MS is widely used for both released glycan profiling and site-specific glycopeptide analysis, providing detailed structural information including branching, fucosylation, and sialylation patterns [94,95].

In glycopeptide workflows, fragmentation strategies such as higher-energy collisional dissociation (HCD) and electron transfer dissociation (ETD) are complementary: HCD provides glycan composition and fragmentation patterns, whereas ETD preserves peptide backbone information, enabling localisation of glycosylation sites. This combination is particularly important for EV glycoproteomics, where biological interpretation often depends on protein-specific glycosylation changes and targeting are often mediated by specific glycoproteins rather than global glycan composition alone [96]. Therefore, glycopeptide-centric LC–MS/MS workflows enable site-specific characterisation of glycosylation on EV proteins. Recent advances in glycoproteomics, including enrichment strategies (lectin affinity, HILIC, hydrazide chemistry) and improved fragmentation methods, have enabled the identification of site-specific glycosylation changes in EV-associated proteins across disease states. These approaches are increasingly used to link glycosylation changes to EV biogenesis, cargo sorting, and biological function.

Matrix-assisted laser desorption/ionisation time-of-flight MS (MALDI-TOF MS) remains a key platform for high-throughput EV glycomics. Its advantages include rapid data acquisition, tolerance to contaminants, and suitability for large cohort studies. MALDI-based workflows are particularly effective for relative glycan profiling and biomarker discovery, although they generally provide less structural depth than LC–MS/MS. Anyway, released glycan analysis is a cornerstone of EV glycomics providing global glycome information. N-glycans are typically enzymatically released using PNGase F, followed by derivatisation (e.g., 2-aminobenzamide labelling or permethylation) to enhance ionisation efficiency and stabilise labile residues such as sialic acids. Advanced derivatisation strategies, including linkage-specific stabilisation (e.g., sialic acid linkage-specific alkylamidation, SALSA), enable discrimination between α2,3- and α2,6-linked sialylation, which is particularly relevant in cancer-associated EV glycomes. MALDI-TOF MS is frequently used in this context due to its robustness and high-throughput capability. Early studies demonstrated that EV-derived N-glycans are enriched in complex-type structures compared with parental cell membranes and exhibit disease- and species-specific features, including differential sialylation and fucosylation patterns [97]. More recent applications include large-scale profiling of urinary EV N-glycomes for biomarker discovery, where MALDI-based workflows enabled detection of hundreds of glycan structures and identification of disease-associated glycosignatures [22]. Beyond oncology, MALDI-TOF MS has been applied to EV analysis in inflammatory, metabolic, and infectious diseases, supporting its versatility as a translational tool for both mechanistic studies and clinical glycomics [98].

Capillary zone electrophoresis coupled with MS (CZE–MS) represents an emerging high-sensitivity platform for EV glycomics, particularly suited for low-input samples. Compared with LC-based approaches, CZE–MS offers exceptional separation efficiency and is well suited for resolving glycan isomers. Recent studies have demonstrated that CZE–MS can detect hundreds of N-glycans from minimal EV material (sub-microlitre plasma equivalents), revealing extensive glycan diversity with high levels of sialylation and fucosylation. Importantly, label-free CZE–MS workflows improve detection of highly sialylated species, which are often underrepresented in derivatisation-based approaches [99,100]. Further methodological advances, including in-capillary enzymatic digestion, enable direct glycan release and analysis within the separation system, reducing sample loss and improving sensitivity. These approaches have achieved deep glycome coverage, including detection of highly sialylated glycans not accessible by conventional workflows [101].

Gas chromatography–MS (GC–MS), particularly in the form of glycan node analysis, provides complementary structural information by quantifying monosaccharide linkages and branching patterns. Because native glycans are non-volatile, GC–MS analysis requires prior chemical derivatisation, typically through hydrolysis, reduction, and peracetylation or permethylation to generate volatile monosaccharide derivatives. These derivatised products enable sensitive detection of glycan linkage patterns, and this approach enables system-level characterisation of glycan features such as fucosylation and branching, which are relevant for EV-mediated organotropism and disease-specific glycosignatures [102].

Overall, MS-based approaches form the analytical backbone of EV glycosylation research. LC–MS/MS provides detailed structural and site-specific information, MALDI-TOF MS enables high-throughput glycan profiling, and CZE–MS offers exceptional sensitivity for low-abundance samples. Chromatographic separation and MS detection are therefore complementary rather than interchangeable. Whereas chromatographic methods reduce sample complexity and improve glycoform resolution, full structural characterisation and site-specific assignment generally require tandem MS workflows. Together with appropriate enrichment and sample preparation strategies, these platforms enable increasingly comprehensive characterisation of EV glycomes across biological and clinical contexts.

### 6.4. Lectin-Based and Affinity Glycan Profiling Assays

Affinity-based approaches, predominantly relying on lectins as glycan-recognising probes, represent a complementary class of analytical methods for EV glycosylation profiling. In contrast to MS-based workflows, these techniques typically analyse intact EVs and preserve the native spatial presentation and multivalency of surface glycans. This enables functional interrogation of glycan–receptor interactions but limits structural resolution to lectin binding specificities. It should be noted that lectin-based platforms provide epitope-level rather than structural information, reflecting lectin binding specificity and avidity rather than precise glycan composition. Despite this limitation, they remain a robust and accessible tool for comparative glycan profiling across EV populations. These platforms are particularly valuable for high-throughput screening, targeting, separation, biomarker discovery, and functional glycomics, where information on relative differences in glycosylation is required [103].

Lectin-based ELISA (often referred to as Enzyme-Linked Lectin Assay, ELLA) is among the most widely used approaches for semi-quantitative profiling of EV surface glycans. In these assays, intact EVs are typically immobilised on microtiter plates and probed with lectins or glycan-binding receptors conjugated to reporters such as horseradish peroxidase or biotin–streptavidin systems. This format enables the detection of major glycan classes, including sialylated, fucosylated, and high-mannose structures, without requiring glycan release or vesicle disruption [104]. Beyond simple profiling, ELLA can be configured in multiple formats. Direct lectin-binding assays enable global glycan profiling of EV preparations, whereas sandwich formats, where EVs are first captured using antibodies (e.g., against CD63 or specific glycoproteins) and subsequently probed with lectins, allow glycoform-specific analysis of defined proteins. In addition, receptor-based ELISA configurations (e.g., using Siglec-Fc constructs) enable functional interrogation of glycan–receptor interactions on EV surfaces. For example, altered lectin binding to EV-associated glycoproteins such as von Willebrand factor has been associated with disease states, including depression, illustrating the diagnostic potential of glycoform-specific EV analysis [54]. In another application, GAGs such as hyaluronan on EVs derived from melanoma cells were quantified using ELISA kit after their enzymatic stripping, revealing differences between non-metastatic and metastatic cell lines and demonstrating the ability of these assays to detect glycan-associated changes in EV composition [105]. ELISA/ELLA are also frequently used to validate glycoengineering strategies, confirming successful modification of EV surface glycans following enzymatic desialylation or synthetic glycan conjugation.

Lectin microarrays represent a high-throughput platform for multiplexed profiling of EV surface glycosylation. These platforms can be implemented in different formats, including classical lectin microarrays, in which multiple lectins are immobilised on a solid surface and interrogated with labelled EVs, as well as glycan or glycoprotein array-type formats, in which glycans, glycoproteins, or EV-associated targets are immobilised and subsequently probed with labelled lectins. In all cases, lectins serve as glycan-recognising probes with defined, although often overlapping, binding specificities, enabling simultaneous interrogation of multiple glycan motifs on intact EVs or EV-associated molecules. Importantly, these approaches preserve the native spatial organisation and multivalency of surface glycans better than released-glycan workflows, allowing biologically relevant glycan–protein interactions to be assessed without full glycan release. Unlike MS-based glycomics, lectin microarrays do not provide detailed structural characterisation of glycans but rather generate glycan-binding signatures reflecting the relative abundance of lectin-recognised epitopes. As such, they are particularly well suited for comparative and sensitive profiling and biomarker discovery, rather than definitive structural analysis [106,107]. These platforms have been successfully applied to distinguish EVs according to their cellular origin, developmental state, and pathophysiological context. For example, lectin-based profiling of EVs derived from induced pluripotent stem cells revealed patterns distinct from non-pluripotent controls, enabling classification of EV populations according to their source and supporting the concept that EV glycosylation reflects cellular identity [108]. In disease-oriented applications, lectin microarray profiling has identified clinically relevant EV glycosignatures. In colorectal cancer, subtype-specific EV glycan patterns enabled dynamic monitoring of tumour progression, highlighting the utility of this approach for longitudinal biomarker studies [31]. Similarly, O-glycan-binding lectins such as Agaricus bisporus agglutinin (ABA) and Amaranthus caudatus agglutinin (ACA) have been used to define EV subpopulations associated with pancreatic cancer, including in patients negative for conventional biomarkers [35]. Lectin microarray profiling has also shown clinical potential in uEV analysis, where disease-associated glycan patterns distinguished autosomal dominant polycystic kidney disease from controls [49]. Beyond descriptive profiling, lectin microarrays can be combined with functional glycomics strategies. Enzymatic modification of EV glycans, particularly desialylation, has been shown to alter lectin-binding profiles and modulate EV uptake by recipient cells in a cell-type-dependent manner, providing mechanistic support for the role of surface glycans in EV–cell interactions [19]. Consistent with this concept, lectin microarray-guided glycoengineering approaches have demonstrated that modulation of EV surface glycosylation can directly influence cellular interactions and uptake efficiency, further supporting the role of EV glycans as functional determinants of intercellular communication rather than passive structural features [109]. In addition, integration of lectin microarray data with machine learning approaches has improved classification performance in glycan-based biomarker studies, further increasing their translational potential [110].

Flow cytometry and fluorescence nanoparticle tracking analysis (F-NTA) with lectin probes provide population-level and, in some settings, near single-particle assessment of EV surface glycosylation. These methods are particularly useful for evaluating EV heterogeneity, quantifying lectin-positive subpopulations, and monitoring how isolation procedures affect surface glycan accessibility. For example, a recent study using lectin-based F-NTA revealed that pre-analytical isolation procedures can significantly alter apparent glycan profiles of EV subpopulations [92].

Lectin-functionalised affinity materials, particularly magnetic beads, are increasingly used to enrich EV subpopulations on the basis of surface glycosylation. In these workflows, lectins such as wheat germ agglutinin (WGA), *Lens culinaris* agglutinin (LCA), or *Solanum tuberosum* lectin (STL) selectively bind EVs bearing compatible glycan motifs. This strategy can simplify sample preparation and enrich glycan-defined vesicle fractions for downstream RNA, protein, or glycan analysis. However, it introduces a selection bias toward lectin-reactive EV subpopulations, which must be taken into account when interpreting results. A clear methodological example was provided by Echevarria et al., who used lectin array screening to identify lectins suitable for the direct isolation of urinary EVs On the basis of this screening, they developed a bead-based capture workflow that enabled uEV isolation without ultracentrifugation, thereby simplifying sample preparation [111]. Lectin-based EV capture has also been applied in biomarker-oriented studies. For example, WGA-conjugated magnetic beads were used to enrich urinary EVs for RNA biomarker discovery in lupus nephritis [112], while LCA- and WGA-coated magnetic beads were used to isolate glycosylated tumour-associated EV fractions in oesophageal squamous cell carcinoma, supporting early diagnostic miRNA signature development [113]. Thus, lectin-based EV capture is best regarded as a targeted enrichment strategy that can either enhance disease-relevant signal or distort population representation, depending on the study objective.

Dot blot-based EV glycan profiling has emerged as a low-complexity and high-sensitivity approach for comparative analysis of EV surface glycans. In this format, EVs are immobilised on membranes and probed with biotinylated lectins, followed by chemiluminescent detection. Because the assay does not require sophisticated instrumentation, it is suitable for rapid screening and translational settings where throughput, cost, and accessibility are important. The DBEG-type approach demonstrated high analytical sensitivity (down to ~10^3^ EVs), applicability across EVs derived from multiple human and rodent cell lines, and the ability to reveal both conserved and cell-specific glycan features [114]. Importantly, this work also highlighted a critical limitation: exogenous glycosylated contaminants can interfere with EV glycan profiling, reinforcing the need for careful sample preparation and interpretation. Accordingly, dot-blot lectin assays are best considered complementary tools for rapid, low-cost glycan profiling, particularly useful for screening and for orthogonal validation of results obtained by MS-based approaches or lectin microarrays.

Affinity- and lectin-based approaches provide a versatile and experimentally accessible framework for EV glycan profiling, particularly suited for comparative analyses, biomarker discovery, and functional interrogation of glycan–receptor interactions. Their key advantage lies in the ability to analyse intact vesicles while preserving glycan spatial organisation and multivalency, which is critical for understanding biologically relevant interactions. In addition, these methods are generally high-throughput, require relatively low sample input, and can be readily adapted for translational and clinical workflows. However, their principal limitation is the lack of structural resolution. Lectin binding reflects glycan epitopes defined by binding specificity and avidity rather than precise glycan composition, linkage, or site-specific context. Furthermore, overlapping lectin specificities and sensitivity to glycan density or accessibility can complicate interpretation. Affinity-based enrichment strategies may also introduce selection bias by preferentially isolating glycan-defined EV subpopulations. Consequently, lectin-based approaches are best considered complementary to MS-based glycomics, providing functional and comparative glycan information that requires orthogonal validation for detailed structural characterisation.

### 6.5. Advanced Biosensing and Nanotechnology-Based Approaches for EV Glycosylation

Advanced biosensing and nanotechnology-based platforms represent a rapidly evolving class of analytical approaches for EV glycosylation. Although many of these systems still rely on lectins or other affinity reagents for glycan recognition, they differ fundamentally from classical lectin-based assays in their analytical architecture, signal transduction mechanisms, and sensitivity. By integrating glycan recognition with physical detection modalities such as plasmonic, magnetic, or time-resolved fluorescence readouts, these platforms enable highly sensitive, low-input, and potentially clinically adaptable EV glycan analysis. More broadly, integrated micro- and nanotechnologies are increasingly being developed for profiling both genetic and modified EV biomarkers, underscoring their translational relevance [115].

Surface plasmon resonance (SPR) is a powerful label-free technique that enables real-time monitoring of biomolecular interactions at sensor surfaces. In EV glycosylation studies, lectin-based SPR is commonly used, where glycan-binding proteins immobilised on gold sensor chips capture EVs based on their surface glycan composition. This allows direct assessment of glycan–lectin interactions and provides quantitative information on binding kinetics and affinity [116,117]. SPR imaging (SPRi) extends this approach by enabling multiplexed detection via spatially resolved sensor arrays, allowing multiple lectins and antibodies to be immobilised simultaneously. This configuration supports parallel glycoprofiling of EV populations and improves analytical throughput. The SPRi system achieves excellent sensitivity for detecting rare glycoproteins on breast cancer-derived EVs [118]. Nanoplasmonic platforms such as nPLEX further increase analytical sensitivity through nanostructured metallic architectures that amplify local plasmonic signals, thereby enabling low-input EV detection and clinically relevant assay times [119].

A particularly relevant recent advance is the self-localized plasmonic nanocavity strategy developed for glycosylation detection of glioblastoma EVs. This work demonstrates that nanocavity-enhanced plasmonic sensing can be adapted specifically to EV glycan analysis, extending beyond generic EV detection toward direct interrogation of glycosylation-associated EV features. This makes it one of the clearest current examples of a nanotechnology-driven glyco-biosensing platform tailored to EVs [120].

SPR-based methods have also been used to detect disease-associated shifts in EV sialylation. Comparative analyses of EVs from benign and malignant prostate cell lines showed increased α2,3- and α2,6-linked sialic acid signatures in cancer-derived EVs, illustrating the ability of plasmonic platforms to detect biologically meaningful glycan changes even when structural depth remains lower than in MS-based approaches [121].

Time-resolved fluorometry using europium nanoparticles (EuNPs) provides a sensitive strategy for detecting glycosylated EV-associated molecules in complex samples. In this format, EVs or EV-associated proteins are first captured, typically through antibodies directed against mucins, integrins, or tetraspanins, and are then detected using lectin- or antibody-functionalised EuNPs. Because europium exhibits a long fluorescence lifetime, time-resolved acquisition reduces background autofluorescence and improves analytical sensitivity. This type of hybrid assay has been applied in colorectal cancer, where combined antibody capture and lectin-based EuNP detection enabled discrimination of cancer-associated EV signatures, including in early-stage disease [32].

Magnetic biosensing offers an alternative strategy for EV glycan analysis, particularly in complex native biofluids. The integrated magnetic analysis of glycans in extracellular vesicles (iMAGE) platform uses lectin-functionalised magnetic nanoparticles to transduce EV glycan binding into measurable magnetic signals through magnetoresistance sensing. A major advantage of this design is that it preferentially detects EV-associated glycans rather than free soluble glycoproteins, thereby improving specificity in biofluids where background glycoproteins would otherwise interfere. The platform also supports multiplexed glycan readouts and is therefore promising for translational diagnostic applications [122].

Taken together, advanced biosensing and nanotechnology-based platforms extend EV glycosylation analysis beyond conventional affinity assays by coupling glycan recognition to highly sensitive physical readouts. Their principal strength lies in low-input, rapid, and potentially clinically adaptable detection, while their main limitation remains the fact that most still provide interaction-based glycan signatures rather than full structural glycomic resolution.

### 6.6. Spectroscopic Techniques

Spectroscopic techniques provide complementary, largely label-free approaches for (EV) analysis, but their contribution to EV glycosylation research remains more limited and generally less structurally resolved than that of MS-based or lectin-array methods. In most applications, these approaches do not identify defined glycan structures directly; instead, they capture composite molecular signatures that may include contributions from glycoconjugates together with proteins, lipids, and nucleic acids [123].

Raman spectroscopy, particularly surface-enhanced Raman spectroscopy (SERS), has been widely used for label-free EV profiling. Their analytical value lies in generating molecular “fingerprints” based on vibrational spectra of EV surface-associated molecules. In this context, carbohydrate-related vibrations can contribute to the overall spectrum, but conventional Raman/SERS usually does not resolve exact glycan composition or linkage. Most published EV Raman studies therefore support classification or fingerprinting rather than true glycomic analysis [124,125].

A more glycan-focused spectroscopic application has recently been demonstrated using lectin-enabled SERS, in which lectin-based nanotags were used to multiplexally profile glycan motifs on the surfaces of cancer-derived sEVs. In that study, glycan signatures changed after glycosidase treatment, supporting the view that SERS can contribute to EV glycan profiling when coupled to selective glycan-recognition chemistry. Even so, the structural depth remains lower than in MS-based glycomics [126].

Nuclear magnetic resonance (NMR) spectroscopy is highly informative for glycan structural analysis in purified systems and can provide quantitative, derivatisation-free information on carbohydrate classes and conformational features. However, because of its relatively low sensitivity and higher sample requirements, its use in EV glycosylation research is currently limited. In practice, NMR is more relevant as a methodological reference point for glycan analysis than as a routine platform for EV glycome characterisation [127].

Overall, spectroscopic techniques are best regarded as complementary in EV glycosylation research. They are useful for rapid, label-free profiling and, in selected cases, glycan-sensitive fingerprinting, but they do not currently replace structurally resolved approaches such as LC–MS/MS, MALDI-based glycomics, or glycopeptide-centric workflows.

### 6.7. Imaging Techniques

Imaging-based approaches provide important spatial and single-particle context for EV analysis and can complement bulk glycomic and affinity-based assays. In EV research, advanced imaging methods are particularly useful for visualising vesicle distribution, uptake, subcellular localisation, and heterogeneity in situ. In the context of EV glycosylation, however, their role remains mainly supportive rather than structurally definitive, because imaging approaches generally do not resolve glycan composition or linkage with the depth achievable by MS-based glycomics [128].

Fluorescence-based imaging can be adapted for glycan-sensitive EV analysis through the use of fluorescently labelled lectins or other glycan-binding probes. Such approaches allow visualisation of glycan-associated signals on EVs and can be combined with protein markers to assess co-localisation and uptake. A representative example is multiplexed immunofluorescent analysis of individual EVs combined with a lectin-binding assay, which enabled interrogation of EV glycan content at the single-EV level and showed that lectin-reactive EV subsets can change under different biological conditions [129].

Electron microscopy (EM), including transmission electron microscopy (TEM) and cryo-electron microscopy (cryo-EM), remains the gold standard for EV morphological characterisation. Although EM does not directly resolve glycan structures, immunogold or lectin-based labelling strategies can be used to visualise glycan-associated epitopes at the vesicle surface. These approaches provide high-resolution spatial information on glycan distribution but remain limited by probe specificity and labelling efficiency [104].

More recently, super-resolution imaging has further expanded this capability. A spectral PAINT-based platform using fluorescent lectin probes enabled multiplexed imaging of individual EVs and mapping of glycosylation patterns together with membrane polarity at the single-particle level, demonstrating pronounced inter- and intraparticle heterogeneity that would not be accessible by bulk methods [130].

Overall, imaging techniques provide valuable complementary information in EV glycosylation research. Their main contribution lies in spatially resolved and single-particle analysis of glycan-associated signals, whereas detailed structural characterisation of EV glycans still requires orthogonal approaches such as MS-based glycomics, glycoproteomics, or lectin-based profiling.

### 6.8. Use of AI and Bioinformatics Tools

The increasing complexity of extracellular vesicle (EV) glycosylation data has driven the development and adoption of advanced bioinformatics and artificial intelligence (AI)-based approaches for data integration, interpretation, and prediction. EV glycomics inherently generates high-dimensional datasets, particularly when combining MS-based glycomics, glycoproteomics, lectin-based profiling, and multi-omics data. Consequently, computational tools are essential for translating these data into biologically and clinically meaningful insights.

At the foundational level, EV-specific databases such as Vesiclepedia [131] and ExoCarta [132] provide curated repositories of EV-associated proteins, lipids, and nucleic acids that can be integrated with glycoproteomic datasets to identify candidate EV glycoproteins and support contextual annotation of glycosylation-related findings. Although these databases do not directly catalogue glycan structures, they are useful for mapping EV-associated molecular cargo and linking glycoproteomic observations to broader EV datasets. More broadly, dedicated EV bioinformatics resources and analysis frameworks support integrated interpretation of EV datasets and facilitate downstream annotation, comparison, and pathway-level analysis [133].

In MS-based EV glycomics and glycoproteomics, specialised bioinformatics tools are required to interpret complex spectra arising from glycan heterogeneity and microheterogeneity. Databases such as GlyTouCan v3.2 [134] provide standardised glycan structure repositories, while software platforms including pGlyco3 v3.1, StrucGP v.1.1.1, and MSFragger-Glyco v4.4 enable site-specific glycopeptide identification, false discovery rate (FDR) control, and assignment of glycan compositions to peptide backbones. These tools integrate multiple fragmentation modes (e.g., HCD and ETD) and advanced scoring algorithms to improve confidence in glycopeptide identification and structural annotation [96,135].

Beyond rule-based annotation, machine learning (ML) and AI approaches are increasingly applied to EV datasets to enable pattern recognition, classification, and predictive modelling. In glycomics, ML has been used to interpret lectin microarray data, classify disease-specific glycosignatures, and integrate glycan features with proteomic and transcriptomic datasets. More broadly, AI-driven glycoinformatics frameworks have been developed to predict glycan structures, biosynthetic pathways, and glycan–protein interactions, addressing key challenges associated with the non-template-driven nature of glycosylation [136].

In the EV field, ML approaches have shown strong potential for biomarker discovery and clinical classification. For example, integrating EV surface marker profiling with ML algorithms such as random forest or support vector machines has enabled high-accuracy classification of disease states, including immune-mediated conditions and cancer. Although most current studies focus primarily on protein markers, incorporating glycan-related features is emerging as a strategy to improve diagnostic performance and capture additional layers of biological information. Furthermore, AI-based integration of EV multi-omics datasets—including proteomics, transcriptomics, and glycomics—enables modelling of intercellular communication networks and glycan-mediated signalling processes. Such integrative approaches are particularly relevant in regenerative medicine, where glycan-dependent interactions (e.g., lectin–glycan and Siglec–glycan signalling) contribute to immune modulation, tissue targeting, and repair processes.

Despite these advances, several challenges remain. Glycomics data are inherently sparse, structurally ambiguous, and less standardised than genomic or proteomic data, which limits the performance and generalisability of AI models. In addition, the lack of large, well-annotated EV glycomics datasets remains a major bottleneck for robust machine learning applications. Continued development of standardised workflows, curated databases, and integrative computational frameworks will therefore be essential for advancing AI-driven research on EV glycosylation. Overall, bioinformatics and AI approaches are becoming indispensable components of EV glycosylation analysis, enabling the integration of complex datasets, improving structural annotation, and supporting the discovery of glycan-based biomarkers and therapeutic targets.

Table 3 summarises the principal analytical approaches currently used in EV glycosylation research, highlighting their analytical targets, information level, key strengths, limitations, and typical applications.

### 6.9. Challenges and Limitations in Studying Glycosylation in EVs

Despite rapid progress, the study of EV glycosylation remains constrained by several conceptual and technical challenges. These arise primarily from the structural complexity of glycans, the intrinsic heterogeneity of EV populations, and the analytical limitations of current glycomic and glycoproteomic workflows. Collectively, these factors complicate biological interpretation, inter-study comparability, and clinical translation [9,103].

A first major challenge is the molecular complexity of glycans themselves. In contrast to nucleic acids or proteins, glycans are not template-driven and can vary extensively in monosaccharide composition, branching, linkage position, stereochemistry, and terminal modifications such as sialylation, fucosylation, or sulfation. As a result, a single EV population may display multiple isomeric and site-specific glycoforms, many of which are difficult to distinguish by mass alone [137]. This is particularly problematic for sialylated glycans, where biologically distinct α2,3- and α2,6-linked isomers may require orthogonal separation strategies, such as ion mobility-based approaches, for reliable discrimination [138]. Glycosylation is also highly dynamic and sensitive to cell state, differentiation, stress, and disease, which further complicates the distinction between biologically meaningful signatures and context-dependent variability.

A second major limitation is the lack of universally standardised workflows for EV isolation, purification, and quality control. Differences in ultracentrifugation, size-exclusion chromatography, precipitation-based methods, affinity capture, and microfluidic isolation strategies can substantially alter EV yield, purity, and detectable glycan composition. This issue is particularly important in glycosylation studies because co-isolated proteins, lipoproteins, and other non-vesicular components are themselves heavily glycosylated and may therefore distort apparent EV glycan signatures. Moreover, the currently used purity metrics, including particle-to-protein ratios and total protein quantification, remain imperfect surrogates and may not reliably distinguish vesicular from non-vesicular material, particularly in complex biofluids. Increasing evidence, therefore, indicates that rigorous orthogonal quality-control approaches, including chromatographic profiling, particle analysis, imaging, and marker validation, are essential prior to downstream glycomic analyses [3,139,140,141].

A third major limitation is the heterogeneity of EV populations. EV preparations typically contain vesicles of different sizes, biogenesis pathways, molecular compositions, and cellular origins, and may also co-isolate abundant non-vesicular contaminants such as soluble glycoproteins, lipoproteins, or urinary polymers. This heterogeneity is a general challenge in EV research, but it is particularly critical for glycosylation analysis because many contaminants are themselves heavily glycosylated and can distort apparent EV glycan profiles [3,139,142]. Recent studies further demonstrate that EV isolation and purification procedures can substantially influence detectable glycan signatures and lectin-binding profiles, highlighting that pre-analytical handling is not analytically neutral and may bias downstream interpretation of EV-associated glycoforms [92,93,140,143]. These findings emphasise the need for rigorous purification workflows, orthogonal quality control, and improved standardisation of EV isolation strategies prior to downstream glycomic analysis.

An additional challenge is the emerging need for single-vesicle glycosylation analysis. Increasing evidence indicates that even highly purified EV preparations contain substantial vesicle-to-vesicle heterogeneity in surface composition and cargo. Although technologies such as nanoflow cytometry, super-resolution imaging, and single-particle analysis are rapidly evolving, current platforms remain limited by low throughput, restricted multiplexing capability, and insufficient sensitivity for low-abundance glycoforms, particularly in small EV subpopulations [144]. Standardisation of isolation procedures, analytical settings, and data-processing workflows will therefore be essential for enabling robust cross-study comparisons.

A particularly important challenge concerns the limitations of current analytical methodologies. MS-based approaches provide the greatest structural depth, but glycan and glycopeptide analysis remains difficult because of low analyte abundance, ionisation bias, incomplete fragmentation, and the need for extensive enrichment and data processing. Highly sialylated or low-abundance glycoforms may be undersampled, and site-specific glycoproteomics remains technically demanding and resource-intensive [96]. Affinity-based methods, including lectin microarrays and lectin capture, offer accessibility and high throughput but provide only epitope-level information and may introduce selection bias due to lectin specificity and avidity. Even when advanced workflows improve sensitivity, for example, in urinary EV glycoproteomics or low-input CZE–MS glycomics, these methods still require careful optimisation and are not yet fully standardised across laboratories [100,101].

Taken together, these limitations indicate that EV glycosylation cannot yet be studied with the same level of routine standardisation as proteomics or transcriptomics. Progress will depend on improved EV purification, broader adoption of orthogonal analytical workflows, better reference materials and databases, and stricter harmonisation of reporting and quality control. Until then, interpretation of EV glycosylation data requires particular caution, especially when comparing studies that differ in sample source, isolation strategy, or analytical platform.

## 7. Future Technologies in EV Glycosylation Research

Future progress in extracellular vesicle (EV) glycosylation research will depend not only on incremental improvements in existing workflows but also on integrating emerging technologies from glycomics, bioengineering, and nanotechnology. These developments are expected to address key current limitations, including low input requirements, structural ambiguity of glycans, EV heterogeneity, and limited translational scalability.

A first important direction is high-resolution glycan analysis. Ion mobility–mass spectrometry (IM–MS) represents a major advance by enabling the separation of glycan isomers and conformers that cannot be resolved by mass-to-charge ratio alone. This is particularly relevant for EV glycosylation, where biologically meaningful differences often arise from subtle linkage or branching variations, especially in sialylated and fucosylated structures [145,146]. Integration of IM–MS with low-input workflows, capillary electrophoresis, and advanced fragmentation strategies is expected to improve structural resolution and reproducibility in EV glycomics.

A second emerging direction is single-cell and ultra-low-input glycomics. Although currently developed primarily for intact cells, these approaches provide a conceptual framework for resolving glycan heterogeneity at high biological resolution. Methods such as scGR-seq and CyTOF-Lec enable simultaneous profiling of glycan features together with transcriptomic or proteomic information, linking glycosylation to cellular identity and function [147,148]. In the context of EVs, such strategies may evolve toward single-vesicle glycomics and spatially resolved glycan mapping, thereby enabling direct correlation between parental cell states, tissue microenvironments, and EV glycan signatures.

A third major area is glycoengineering of EVs, which shifts the field from descriptive analysis toward functional manipulation. Reconfigurable engineering of the EV glycocalyx has demonstrated that defined glycan motifs can be engineered onto EV surfaces to enhance cell-specific targeting and uptake [88]. More recently, CRISPR/Cas9-based engineering of parental cells has enabled the generation of “simple-cell” systems with controlled glycosylation pathways, resulting in EVs with predictable and reproducible glycan profiles [149]. Emerging glycoengineering strategies further suggest that selective modification or depletion of specific glycan classes on EV surfaces may substantially alter vesicle uptake, cargo delivery, and cellular tropism, highlighting the functional importance of the EV glycocalyx in therapeutic engineering [150]. Such approaches may provide powerful experimental platforms for dissecting glycan function and for designing EV-based therapeutics with optimised biodistribution and targeting.

A fourth direction is the increasing role of advanced biosensing and nanotechnology-based platforms. As discussed in the preceding Section 6.5, emerging platforms such as nanoplasmonic sensors, magnetic glycan detection systems, and time-resolved fluorescence assays enable highly sensitive, low-volume, and rapid detection of EV glycan signatures. Recent developments, including plasmonic nanocavity-based sensing strategies, further demonstrate that these technologies can be adapted for glycosylation-specific EV analysis rather than generic EV detection [120]. Importantly, these platforms are increasingly integrated into microfluidic and lab-on-a-chip systems, enabling multiplexed, clinically scalable EV analysis. In this context, advanced biosensing technologies are expected to play a central role in translating EV glycosylation from a research tool into a diagnostic and therapeutic monitoring modality [115]. Unlike classical glycomic workflows, which prioritise structural resolution, these systems emphasise sensitivity, speed, and clinical applicability, thereby complementing rather than replacing MS-based approaches.

A fifth key direction is multi-omics integration. EV glycosylation does not act in isolation but is tightly linked to protein composition, lipid organisation, and cellular origin. Multi-omics approaches, therefore, enable integrated analysis of EV-mediated communication, biomarker discovery, and disease mechanisms. Increasingly, computational frameworks are being developed to combine glycomic, proteomic, and transcriptomic data into unified models of EV biology [151]. Emerging evidence further suggests that glycosylated RNAs (glycoRNAs) may represent an additional layer of EV-associated molecular complexity, although their biological and translational relevance remains incompletely understood [152].

Taken together, these emerging directions indicate that EV glycosylation research is transitioning from descriptive glycan profiling toward an integrated and translationally oriented discipline. Advances in structural glycomics, single-particle analysis, glycoengineering, biosensing, and multi-omics integration are likely to converge, enabling precise manipulation, detection, and functional interpretation of EV glycans. This convergence will be essential for unlocking the full potential of EV glycosylation in diagnostics, targeted delivery, and regenerative medicine.

## 8. Biosafety and Barriers to Clinical Translation

The clinical development of EV-based therapeutics, including glycoengineered EVs, is constrained not only by analytical and manufacturing challenges but also by biosafety requirements. These considerations are closely interlinked and are best viewed within a single translational framework. Although EVs are often regarded as biocompatible and potentially less immunogenic than cell-based therapies, their safety profile depends critically on the source material, manufacturing process, purification strategy, and final product characterisation.

A first major concern is biological contamination. EV preparations derived from human or animal cells, tissues, or biofluids may carry adventitious agents, including viruses, mycoplasma, endotoxins, and other microbiological contaminants. Additional risk may be introduced during cell culture, particularly when undefined supplements such as fetal bovine serum or human platelet lysate are used, because these materials may contain xenogeneic vesicles, infectious agents, or confounding bioactive components. These issues are especially important for therapeutic EV products, where biosafety expectations extend beyond research-grade characterisation and require traceable source materials, viral safety assessment, and contamination control under GMP-compatible conditions [153,154,155].

A second concern is product heterogeneity and compositional inconsistency. EVs are intrinsically heterogeneous with respect to size, biogenesis, cellular origin, and molecular cargo. This heterogeneity is amplified in therapeutic manufacturing, where batch-to-batch variability may arise from donor differences, cell passage number, culture conditions, isolation method, and storage. For glycosylation-focused applications, this problem is particularly acute because EV surface glycan composition is highly sensitive to cell state and process conditions. Consequently, clinically relevant glycan signatures may be difficult to reproduce unless upstream manufacturing and downstream purification are rigorously standardised [3,154].

A third barrier is the lack of robust quality and potency frameworks. For clinical translation, EV products must be defined not only by particle counts or canonical markers but also by critical quality attributes linked to mechanism of action, purity, stability, and safety. In glycoengineered EVs, this requirement becomes even more demanding, because the therapeutic effect may depend on controlled surface glycosylation, receptor targeting, or altered biodistribution. This means that release criteria for future EV therapeutics may need to include not only conventional EV characterisation but also glycan-sensitive analytical controls. At present, however, such standards are not yet harmonised across the field [3,154].

A fourth translational limitation is regulatory and manufacturing scalability. Clinical-grade EV production requires traceable source materials, validated purification workflows, reproducible large-scale manufacturing, and stability control throughout storage and distribution. These requirements are challenging for native EVs and become even more complex for engineered products, where additional modifications such as cargo loading, membrane engineering, or glycoengineering must be shown to be reproducible, safe, and functionally justified. Recent reviews and manufacturing frameworks emphasise that EV therapeutics must be evaluated within GMP-oriented pipelines analogous to other biological medicinal products, including quality control, sterility, viral safety, storage stability, and clinical-grade batch release [154,155].

Within this context, engineered EVs should be viewed both as an opportunity and as an additional regulatory challenge. Tailoring EV glycosylation, cargo composition, or membrane surface can improve targeting, biodistribution, immunomodulation, and regenerative efficacy, potentially helping overcome some limitations of native EV preparations. At the same time, such modifications introduce new questions regarding off-target interactions, altered immune recognition, long-term safety, and manufacturing reproducibility. Thus, engineering does not eliminate translational barriers; rather, it shifts them toward more stringent requirements for product definition and quality assurance.

Taken together, the major barriers to clinical translation of EV glycosylation-based therapeutics are not solely biological or technological but integrative, such as safe source selection, contamination control, reproducible manufacturing, glycan-sensitive quality control, and regulatory harmonisation, which must all be addressed simultaneously. Progress in this area will depend on the convergence of standardised EV guidelines, GMP-compliant production strategies, and analytically robust definitions of EV identity and potency.

## 9. Conclusions

Glycosylation represents a key functional layer of EV biology, influencing vesicle stability, biodistribution, cellular targeting, and immune interactions. Through glycan–receptor recognition, including lectin- and Siglec-mediated pathways, EV surface glycans contribute to intercellular communication and tissue-specific targeting, positioning glycosylation as an active regulator rather than a passive structural feature.

EV glycosylation has emerged as a promising source of disease biomarkers, with the strongest evidence currently in oncology, where tumour-specific glycosignatures have been identified across multiple cancer types. In other disease areas, including neurological, metabolic, and inflammatory conditions, glycan-based EV profiling remains less developed but shows growing potential as a complementary diagnostic modality.

In regenerative medicine, EV glycosylation plays a critical role in modulating tissue targeting, extracellular matrix interactions, and immune responses. While most current evidence remains indirect, glycoengineering approaches demonstrate that controlled modification of EV surface glycans can enhance therapeutic delivery and efficacy, supporting the translational relevance of EV glycosylation across multiple tissues.

Advances in analytical technologies—including MS-based glycomics, lectin-based platforms, biosensing systems, and computational tools—have significantly expanded the ability to characterise EV glycomes. However, major challenges remain, particularly related to glycan structural complexity, EV heterogeneity, pre-analytical variability, and limited standardisation across methodologies.

Future progress will depend on integrating high-resolution glycoanalytics, single-particle approaches, AI-driven data analysis, and multi-omics strategies, along with the development of reproducible, clinically compliant EV production workflows. Although clinical translation is still at an early stage, EV glycosylation holds substantial promise as a bridge between fundamental EV biology, precision diagnostics, and targeted regenerative therapeutics.

## Figures and Tables

**Table 1:** Disease-associated changes in EV glycosylation, evidence level, and biomarker potential.

Disease	EV Glycosylation-Related Change	Evidence Level	Biomarker Potential	Key Reference(s)
Lung cancer	Subtype-specific N-glycans, integrin glycoforms; increased sialylated-fucosylated N-glycans and site-specific glycan heterogeneity in plasma EVs	Preclinical/clinical	Diagnostic/ stratification	[24,27]
Hepatocellular carcinoma	Differential N-glycopeptides, site-specific glycosylation changes in uEVs	Clinical	Diagnostic	[30]
Colorectal cancer	Differences in EV glycan signatures; glycan-defined EV subpopulations	Preclinical	Diagnostic/ prognostic	[31,32]
Pancreatic cancer	O-glycan–associated EV subpopulations in serum EVs	Clinical	Diagnostic/ monitoring	[35]
Bladder cancer	Decreased fucosylation, increased sialylation, altered N-glycan diversity in uEVs	Clinical	Diagnostic	[22]
Ovarian cancer	N-glycans composition	Preclinical	Diagnostic/ stratification	[37,38,39]
Prostate cancer	Altered uEV N-glycans	Clinical	Diagnostic	[41]
Polycystic kidney disease	Altered glycosylation profiles of uEVs	Clinical	Exploratory/ diagnostic	[49]
Neurological/ neuropsychiatric disorders	Serum EVs N-glycan signatures in epilepsy; altered plasma EV-associated vWF glycosylation in depression	Clinical	Diagnostic/ stratification	[53,54]
Musculoskeletal/ joint diseases	EV-associated glycoprotein changes	Indirect	Exploratory	[62,63]

**Table 2:** Functional roles of EV glycosylation in regenerative contexts.

Regenerative Context	Glycosylation-Related Mechanism	Functional Effect	Evidence Level	Key Reference(s)
Renal regeneration	Hyaluronic acid-mediated targeting via CD44/TLR4	Kidney targeting	Preclinical	[77]
Liver regeneration	Desialylation-enhanced uptake via ASGPR	Hepatic tropism	Preclinical	[78]
Skin/wound regeneration	FUT7-mediated glycoengineering enhances EV targeting	Wound repair	Preclinical	[87]
Angiogenesis/vascular regeneration	Glycocalyx engineering with defined glycan ligands (e.g., sLeX)	Endothelial targeting	Preclinical	[88]
Immunomodulation in regeneration	Siglec-mediated glycan/glycolipid recognition	Immune modulation	Preclinical	[89]

**Table 3:** Key analytical approaches for EV glycosylation research.

Method	Analytical Target	Information Level	Key Strengths	Key Limitations	Typical Applications
LC–MS/MS	Glycans, glycopeptides	Structural/ site-specific	High resolution	Complex workflow	Glycome profiling
MALDI-TOF MS	Glycans	Compositional	Glycan composition	Lower depth	Biomarker screening
CZE–MS	Glycans	Structural	Low-input sensitivity	Technical complexity	Low-input glycomics
GC–MS (glycan node analysis)	Glycan motifs	Linkage-level	Branching insight	No intact glycans	Motif analysis
Lectin microarray	Surface glycans	Epitope-level	High throughput; intact EVs	Low structural depth	Comparative profiling
Lectin-ELISA/ELLA	Surface glycans, glycoforms	Epitope-level	Accessible; targeted	Probe bias	Targeted validation
Lectin-based EV capture	Glycan-defined EV subsets	Enrichment-level	Subset enrichment	Selection bias	Biomarker enrichment
SPR/ nanoplasmonic biosensing	Glycan interactions	Interaction-level	Label-free; sensitive	Limited structure	Real-time profiling
Magnetic glycan sensing (iMAGE)	EV-bound glycans	Interaction-level	Native biofluids	Emerging platform	Rapid profiling
AI/ bioinformatics tools	Glyco-omics datasets	Integrative	Data integration	Sparse datasets	Biomarker modelling

## Data Availability

No new data were created or analysed in this study. Data sharing is not applicable to this article.
